# Richmond Medical Journal

**Published:** 1868

**Authors:** 


### RICHMOND MEDICAL JOURNAL.

We received, some weeks ago, a communication from Dr. E. S. Gaillard in reference to the removal of the "Richmond Medical Jonrnal" from Richmond to Louisville, Ky., where it will be published under the name of the "Richmond and Louisville Medical Journal."

As our Journal goes to press, we discover that the communication has been mislaid.

We bear willing testimony to the ability of the Richmond Medical Journal under the editorial management of Dr. Gaillard, and shall always welcome it to our office.

Dr. G., we are informed, accepts a Chair in the Kentucky School of Medicine.

We are requested by the Editor of this popular and useful Medical Journal to state that our subscribers can receive it regularly at $3 a year. This is certainly a reduced price for so large and valuable a monthly periodical, and will doubtless add greatly to its circulation. The regularly published rates require $5 per annum, in advance.

